# Toward Inclusive Approaches in the Design, Development, and Implementation of eHealth in the Intellectual Disability Sector: Scoping Review

**DOI:** 10.2196/45819

**Published:** 2023-05-30

**Authors:** Julia F E van Calis, Kirsten E Bevelander, Anneke W C van der Cruijsen, Geraline L Leusink, Jenneken Naaldenberg

**Affiliations:** 1 Department of Primary and Community Care Radboud University Medical Center Nijmegen Netherlands

**Keywords:** eHealth, digital health, intellectual disability, inclusive research, involvement, participatory development, scoping review, Centre for eHealth Research and Disease management, CeHRes roadmap, Nonadoption, Abandonment, and challenges to the Scale-up, Spread, and Sustainability framework, NASSS, review method, inclusive, inclusivity, accessibility, participatory, iterative, design, develop, intellectually disabled

## Abstract

**Background:**

The use of eHealth is more challenging for people with intellectual disabilities (IDs) than for the general population because the technologies often do not fit the complex needs and living circumstances of people with IDs. A translational gap exists between the developed technology and users’ needs and capabilities. User involvement approaches have been developed to overcome this mismatch during the design, development, and implementation processes of the technology. The effectiveness and use of eHealth have received much scholarly attention, but little is known about user involvement approaches.

**Objective:**

In this scoping review, we aimed to identify the inclusive approaches currently used for the design, development, and implementation of eHealth for people with IDs. We reviewed how and in what phases people with IDs and other stakeholders were included in these processes. We used 9 domains identified from the Centre for eHealth Research and Disease management road map and the Nonadoption, Abandonment, and challenges to the Scale-up, Spread, and Sustainability framework to gain insight into these processes.

**Methods:**

We identified both scientific and gray literature through systematic searches in PubMed, Embase, PsycINFO, CINAHL, Cochrane, Web of Science, Google Scholar, and (websites of) relevant intermediate (health care) organizations. We included studies published since 1995 that showed the design, development, or implementation processes of eHealth for people with IDs. Data were analyzed along 9 domains: participatory development, iterative process, value specification, value proposition, technological development and design, organization, external context, implementation, and evaluation.

**Results:**

The search strategy resulted in 10,639 studies, of which 17 (0.16%) met the inclusion criteria. Various approaches were used to guide user involvement (eg, human or user-centered design and participatory development), most of which applied an iterative process mainly during technological development. The involvement of stakeholders other than end users was described in less detail. The literature focused on the application of eHealth at an individual level and did not consider the organizational context. Inclusive approaches in the design and development phases were well described; however, the implementation phase remained underexposed.

**Conclusions:**

The participatory development, iterative process, and technological development and design domains showed inclusive approaches applied at the start of and during the development, whereas only a few approaches involved end users and iterative processes at the end of the process and during implementation. The literature focused primarily on the individual use of the technology, and the external, organizational, and financial contextual preconditions received less attention. However, members of this target group rely on their (social) environment for care and support. More attention is needed for these underrepresented domains, and key stakeholders should be included further on in the process to reduce the translational gap that exists between the developed technologies and user needs, capabilities, and context.

## Introduction

### Background

The number of eHealth applications has increased exponentially over the last years. In general, eHealth can be defined as “the use of technologies to improve health, well-being, and healthcare” [1]. Nonetheless, the implementation of eHealth technology remains challenging and often cannot be sustained over time [2-4]. The use of eHealth is more challenging for people with intellectual disabilities (IDs) compared with the general population, as the technologies often do not fit the complex needs and living circumstances of people with IDs [5,6]. In the rapidly changing digital environment, people with IDs often need support when using eHealth because they experience difficulties in acquiring digital literacy skills and using digital devices or the internet [7-9]. These difficulties show the translational gap that exists between the developed technology and these users’ needs and capabilities, although there are approaches available to overcome this mismatch [10-12], for example, by involving users in the development and implementation processes of the technology. However, more knowledge is needed on these inclusive eHealth trajectories. Therefore, this scoping review examined how inclusive approaches have been used in the design, development, and implementation of eHealth for people with IDs.

IDs can be defined as considerable limitations in both intellectual functioning and adaptive behavior as expressed in conceptual, social, and practical adaptive skills [13]. People with IDs have heterogeneous needs for health and support and are strongly dependent on their social environment for access to, and use of, eHealth [3]. This dependency also causes struggles in communication, personal care, traveling, and living [14]. Reviews by Burke [15] and Vázquez et al [16] showed that eHealth has the potential to increase the level of independence of people with IDs and to support their higher demand for personalized care [15,16]. However, technological innovations are often too complex for people with IDs to use independently [10,11]. One explanation is that eHealth is often developed and implemented without the involvement of key stakeholders, such as people with IDs and their caregivers and care provider organizations that use the eHealth applications [1,17]. Including these stakeholders in the development and implementation of eHealth ensures that eHealth is adjusted to their living environment and needs for health and support, thereby increasing the sustainability of eHealth use over time [1,18,19]. This can be achieved by applying inclusive research and design and giving end users and key stakeholders an active role as experiential experts, co-designers, or coresearchers throughout the process [20-22].

Several approaches can be applied to technology design, development, and implementation. For example, design thinking is used to explore the context of complex problems and generate solutions in an iterative process by keeping the users’ needs central [23]. Universal design aims to maximize usability by individuals with a wide variety of characteristics by applying 7 principles (eg, equitable use, flexibility in use, and perceptible information) [24]. Another example is the Consolidated Framework for Implementation Research, which has been developed to guide the systematic assessment of implementation, formative evaluations, and the identification of factors that might influence intervention implementation [25,26]. Although these approaches are widely used in practice, they do not focus specifically on health care–related technologies [26,27]. Frameworks that focus on such technologies (eg, the Health Technology Assessment–inspired Model for Assessment of Telemedicine applications and the eHealth value model) [28,29] provide evaluation tools to assess the value and effectiveness of health care technologies but only marginally give practical guidance on inclusive design, development, and implementation.

The Centre for eHealth Research and Disease management (CeHRes) road map is an example of this and is based on existing evidence-based models, frameworks, and methods such as participatory development and business modeling. This road map can be used to guide the development, implementation, and evaluation of eHealth technologies [1,17]. Another example is the Nonadoption, Abandonment, and challenges to the Scale-up, Spread, and Sustainability (NASSS) framework, which reviews the implementation of health care technology in multiple domains (eg, technology, value proposition, adopters, and organization) [30]. According to the NASSS framework, the development of technology is a never-ending process in which the technology can be adjusted to fit each specific setting and context [31]. The NASSS framework has been widely applied in eHealth research and extended with the practical NASSS-Complexity Assessment Tool (NASSS-CAT) [32-35]. Both the CeHRes road map and the NASSS framework assess eHealth technology in which iterative processes play a central role in the design, development, and implementation while involving end users and other key stakeholders [1,30].

The term *eHealth* is broad and has various definitions [36]. For example, Oh et al [36] described it as “characterizing not only a technical development, but also a state of mind, a way of thinking, an attitude, and a commitment for networked, global thinking, to improve health care locally, regionally, and worldwide by using information and communication technology,” whereas Eysenbach [37] described it “as the cost-effective and secure use of information and communications technologies in support of health and health-related fields, including health care services, health surveillance, health literature, and health education, knowledge, and research.” In this study, we specified the general definition to the context of people with IDs who often live within health care organizations or assisted living facilities [1]. Therefore, we did not focus on technologies with an educational purpose without health, well-being, or health care–related content or medical technologies such as hospital equipment and implanted devices.

### Objective

In this scoping review, we aimed to identify the inclusive approaches that were used for the design, development, and implementation of eHealth for people with IDs. In addition, we reviewed how and in what phases people with IDs and other stakeholders were included in the process. We used components identified from the CeHRes road map and NASSS framework to examine the current literature on eHealth design, development, and implementation processes.

## Methods

### Study Design

We used the scoping review methodology that is proposed by Levac et al [38] and is based on the framework developed by Arksey and O’Malley [39] to guide the review process. This methodology consists of five stages: (1) identifying the research questions; (2) identifying relevant studies; (3) selecting relevant studies; (4) charting the data; and (5) collating, summarizing, and reporting the results [38].

### Identifying Research Questions

The objective was divided into four subquestions: (1) What theories or frameworks are used in the design, development, and implementation of eHealth for people with ID? (2) Who is involved in the process of eHealth design, development, and implementation for people with ID? (3) In what phases and activities of eHealth design, development, and implementation are people with ID and stakeholders involved? and (4) What components from the CeHRes road map and the NASSS framework can be identified in the design, development, and implementation of eHealth for people with ID?

### Identifying Relevant Studies

A search string was developed with assistance from an information expert, using the Population, Intervention, Comparison, and Outcome approach [40]. The following 7 databases were searched: PubMed, Embase, PsycINFO, CINAHL, Cochrane, Web of Science, and Google Scholar. Multimedia Appendix 1 [1,13,41-43] shows the full search strings used for PubMed, consisting of blocks with terms describing “intellectual disability” [AND] “eHealth” [AND] “design” [OR] “development” [OR] “implementation,” which was then adopted for each subsequent database. The terms design, development, and implementation were connected by [OR] to search for a combination of the phases in which the process was described or the studies that described them separately. Gray literature, peer-reviewed reports, and non–peer-reviewed reports, such as Dutch unpublished documentation, were included by contacting 2 intermediate organizations that share the knowledge of producers with knowledge users and 7 care organizations for people with IDs via email for (unpublished) literature. The websites of relevant (health care) organizations were also examined for documentation. Additional articles were identified by manually searching the reference lists of the included articles, including searching for previous or follow-up articles of the included articles.

### Selecting Relevant Studies

The PRISMA-ScR (Preferred Reporting Items for Systematic Reviews and Meta-Analyses extension for Scoping Reviews) [44] guided the selection process (Table 1). The search included literature published between January 1995, when the internet is first introduced in health care [35], and January 2022. Studies were included if at least 1 of the 3 process descriptions (ie, design, development, or implementation) was present in the article. Both title and abstract and full-text screenings were performed by 1 researcher (JFEC). If the inclusion of a title and abstract was unclear, it was included in the full-text screening and reviewed by another independent reviewer (KEB) in 3 eligibility stages.

**Table 1:** Inclusion and exclusion criteria.

Criteria	Inclusion	Exclusion
Type of studies	All full-text studies (eg, articles, dissertations, conference papers, reports, and gray literature from [health care] organizations)	Abstracts and studies presenting only psychometric data
Period	From January 1, 1995, to January 31, 2022	Before January 1, 1995, and after January 31, 2022
Language	English or Dutch	All other languages
Population	People with intellectual or developmental disability	People with cognitive disabilities caused by traumatic brain injury, stroke, cancer (treatment), or dementia
Intervention	Technology that is created to improve health or well-being or health care related (eg, technology to provide support with medication intake or daily [independent] living)	Educational application of the technology without health, well-being, or health care–related content and medical application of the technology (eg, hospital equipment, such as heart monitors, and implanted devices, such as pacemakers)
Outcome	The design process of eHealth interventions for people with intellectual disabilities The (technological) development process of eHealth interventions The implementation process of eHealth interventions for people with intellectual disabilities	Focus on the design (appearance [eg, colors, visuals, and font style]) of eHealth without describing the process Focus on the development (eg, content) of eHealth without describing the process Focus on the use or effectiveness of eHealth interventions after implementation

### Data Charting and Collating, Summarizing, and Reporting the Results

First, the following key information was extracted from each paper: authors, title, year of publication, origin or country, article type, aim or purpose, study population (target end user population), sample size, methodology, and intervention type (purpose of the eHealth) [1]. Next, the theories, frameworks, and approaches used in the design, development, and implementation were extracted. Moreover, the people involved and how and in what phases or activities they were involved were reviewed. The extraction of the design, development, and implementation processes was guided by 9 domains identified from the CeHRes road map and NASSS framework, as described in the following paragraph.

The CeHRes road map describes clear development activities and combines participatory development, human-centered design, business modeling, and persuasive design in 5 intertwined phases and connecting cycles (formative evaluations): contextual inquiry, value specification, design, operationalization, and summative evaluation [1]. The NASSS framework studies the complexity of 7 domains: the condition (ie, the nature of the condition, sociocultural factors, and comorbidities); technology; value proposition; adopters; organization; wider system; and embedding and adaptation over time [30]. The NASSS framework emphasizes that the technology needs to fit each specific setting and context and shows important preconditions for implementation. After combining the phases from the CeHRes road map and the domains from the NASSS framework, the following nine main domains with their corresponding components were defined for this study:

1. Participatory development: the approach actively involves all stakeholders in the development process to help ensure that the result meets their needs and is usable [1,17]. This includes cocreation, multidisciplinary project management, and the inclusion of stakeholders’ perspectives.
2. Iterative process: continuous evaluations are performed during the design, development, and implementation of the technology. The evaluation is interwoven with all stages in the development process [1], including continuous evaluation and checking whether the outcomes of the previous phases are accounted for.
3. Value specification: creating the optimum level of return for end users and other stakeholders involved by identifying, analyzing, gathering, and mapping their values, for example, easy-to-read text and accessibility of the technology [1,17]. This consists of end users, conditions or illnesses, sociocultural factors, stakeholder identification, stakeholder analysis, and value identification.
4. Value proposition: this involves explicating the value that the technology might generate for different groups of people. Values can be financial, such as profit, or nonfinancial, such as control of symptoms [1,45]. This includes the business model, the supply and demand model, and ownership.
5. Technological development and design: describing the technology (eg, a tool or piece of software) and how it might affect care [30], this includes technology requirements, prototyping (lo-fi and hi-fi), and usability tests.
6. Organization: considering the changes needed for the (health care) organizations to implement and use the technology and the consequences of the technology after it is introduced [30,35], this covers the capacity and readiness to innovate, nature of adoption and funding decisions, and changes in organizational routines.
7. External context: external conditions that could complicate the adoption and spread of the technology [27], including the political and policy context, regulatory and legal issues, professional bodies, and interorganizational networking, are considered.
8. Implementation: this includes developing an implementation plan with a set of conditions or activities designed to start using technology in practice [1] and discovering whether the implementation is accounted for from the start and determining activities for the implementation plan.
9. Evaluation: this includes understanding the relative benefits and costs of the technology in the context of the proposed implementation [1,30], determining the impact on the context and stakeholders, and analyzing the uptake of the technology.

Multimedia Appendix 2 shows a table with the 9 domains and their corresponding descriptions used for data extraction. The data extraction chart table was created iteratively based on feedback from the authors and a sounding board consisting of coresearchers and eHealth project managers from disability health care organizations. A test analysis was performed on 3 studies by using the first version of the data extraction chart table. The test analysis was used to refine the data extraction chart table. The Results section covers these 9 domains, following the research questions specified in the Identifying Research Questions section.

## Results

### Article Selection

**Figure 1:**
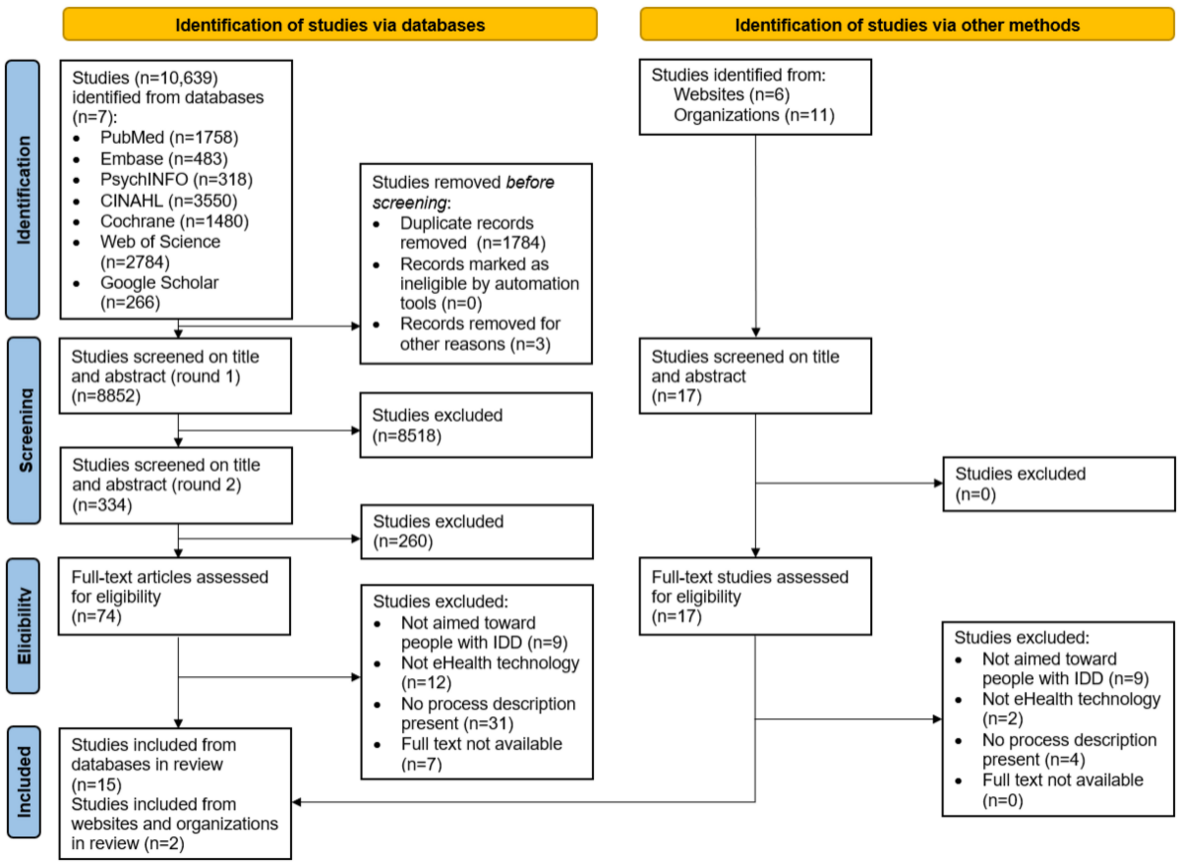
PRISMA-ScR (Preferred Reporting Items of Systematic Reviews and Meta-Analyses extension for Scoping Reviews) flowchart. IDD: intellectual and developmental disability.

The identification phase resulted in 10,639 records. There were 1784 duplicates, and these together with 3 studies from before 1995 were removed, leaving 8852 (83.20%) studies. Title and abstract screening was performed using the inclusion and exclusion criteria (Table 1). In the screening phase, the main reasons for exclusion were that the target group did not fit our criteria and that the technology did not match the definition of eHealth used in this study. The absence of a description of how the eHealth was developed, designed, or implemented (referred to as process description in this study) was the main reason for exclusion in the eligibility phase. Of the 8852 studies, 8778 (99.16%) studies were removed, resulting in 74 (0.84%) studies whose full texts were read by the first author (JFEC) to screen for inclusion. In the event of doubt, the studies were read by another independent reviewer (KEB). After the full-text screening, 15 (20%) of the 74 studies were eligible for inclusion. The same method was applied to the gray literature, in which 2 studies were included, leaving a total of 17 studies included (Figure 1).

### Study Summary

An overview of information obtained from the included studies is presented in Table 2. Table 2 shows the distribution of the studies across the continents. Of the 17 studies, most studies (n=10, 59%) were conducted in Europe, 4 (24%) studies were performed in the United States, and the remaining 3 (18%) studies were conducted in Australia. These studies included a variety of topics and purposes of eHealth technologies, such as supporting and training life skills and communication. End users of the eHealth technologies included, for example, adults with intellectual and developmental disabilities and people with IDs and other impairments such as sensory and speech impairments.

**Table 2:** Overview of information from the included studies—source and country; the identified eHealth purposes; the approaches, theories, or frameworks used; the processes presented; and the targeted end users (n=17).

Source, country	Purpose of eHealth	Approach, theory, or framework	Design, development, or implementation	Targeted end users
Bayor [46], 2019, the United States	Support life skills development	Competency-based design and participatory action research with collaborative technology workshops	Design and development	Young adults with IDs^a^
Brown et al [47], 2011, the United Kingdom	Skill development for route learning	Phased development and implementation with user-sensitive inclusive design	Design, development, and implementation	People with IDs and additional sensory impairments
Brown et al [48], 2016, Australia	Learning and training life skills	Iterative co-design processes by Brereton et al [49], 2015	Design, development, and implementation	Adults with IDs
Davies et al [50], 2015, the United States	Accessible interface for Facebook	Iterative design and development	Development	Adults with IDs
Dekelver et al [51], 2015, Belgium	Traveling independently	Human-centered design: methods to support human-centered design (Maguire) [52], 2021, 10 heuristics for user interface design (Nielsen) [53], 1994, and designing for users with cognitive disabilities (Friedman and Bryen) [54], 2007	Development	People with IDs
Dekelver et al [55], 2015, Belgium^b^	Accessible mobile apps	Human- or user-centered design with persona and WAI^c^ guidelines	Design and development	People with IDD^d^
Duval et al [56], 2018, the United States	Speech articulation therapy	Iterative user-centered design	Design and development	Adults with developmental disabilities co-occurring with speech impairment
Engler and Schulze [57], 2017, Germany	Managing daily activities independently	User-centered design	Design	People with Down syndrome
Furberg et al [58], 2018, the United States	Decision support	Feature-driven design approach and user-centered design process	Design and development	Individuals with Fragile X syndrome
Igual et al [59], 2014, Spain	Living independently	Requirement’s engineering	Development	People with IDs and older people
Kaimara et al [60], 2021, Greece	Daily living skills training	5W2H^e^ framework and participatory design	Design and development	Children with SENs^f^
Kerkhof et al [19], 2017, the Netherlands	Structure and support for daily activities	Participatory design and iterative process	Development and implementation	Clients with IDs
Kranenborg et al [61], 2013, the Netherlands	Accessible user interfaces	Situated cognitive engineering	Design and development	People with IDs
Lennox et al [62], 2009, Australia	Accessible web-based learning	W3C^g^ guidelines—accessibility guidelines double A	Design and development	Adults with IDs and diabetes
Read et al [63], 2013, the United Kingdom	Support in case of a bereavement	Participatory action research	Design and development	People with IDs
Robb et al [64], 2019, Ireland	Cognitive training game	Participatory design	Design and development	Children with a rare genetic syndrome linked to ID
Wilson et al [65], 2016, Australia	Support with communicating	RAID^h^ process	Design and development	Young adults with IDs

**^a^**ID: intellectual disability.

^b^To separate and identify the two studies from the same author and year of publication.

^c^WAI: Web Accessibility Initiative.

^d^IDD: intellectual and developmental disability.

^e^5W2H: What, Where, When, Who, Why, How, and How much.

^f^SEN: special educational need.

^g^W3C: World Wide Web Consortium.

^h^RAID: Reflective Agile Iterative Design.

### Domains

We identified 10,639 studies with our search strategy. Of these identified studies, only 17 (0.16%) provided a process description of eHealth design, development, or implementation for people with IDs, and these were analyzed based on the 9 domains. Regarding the first domain, 14 (82%) of the 17 studies [19,46-48,51,55-58,60,62-65] applied participatory development by involving end users and other stakeholders in different phases. For the second domain, 13 (76%) of the 17 studies [19,46-48,51,56,58,60-65] performed an iterative process through continuous evaluations, the use of prototypes, and the retrieval of user requirements. The third domain was reflected in 11 (65%) of the 17 studies [19,46,50,51,56-58,61,62,64,65], which performed a value specification or part of it, such as the description of end users’ characteristics and the identification of other stakeholders besides end users. Similarly, in 7 (41%) of the 17 studies [47,48,55,59,60,62,63], only small parts were written about the fourth domain, value proposition, such as the values retrieved and the origin of these values. In all (17/17, 100%) studies, information on the fifth domain regarding technological development and design was provided, in which the development of prototypes based on requirements and testing of the prototypes was described. Only 1 (6%) of the 17 studies [19] provided information about the sixth and seventh domains concerning the organization and the external context. In total, 7 (41%) of the 17 studies [19,46-48,50,56,65] referred to the eighth domain, implementation, with implementation accounted for from the start and future implementation mentioned. The last and ninth domain, evaluation, was mentioned by 9 (53%) of the 17 studies [46,51,56,57,59-61,63,65] describing uptake and 7 (41%) of the 17 studies [19,48,50,57,58,65] showing impact.

### Theories, Frameworks, and Approaches Used (Domains 1 and 2)

#### Overview

This section presents 2 domains covering theories: frameworks and approaches from the included papers that were intertwined with participatory development (domain 1) and iterative processes (domain 2; Table 2 provides an overview). Various inclusive theories and frameworks were used such as the sensitive inclusive design approach [47], human- or user-centered design [51,55-58], participatory design [19,60,64], participatory action research [46,63], and co-design [46,48,65]. The iterative approach was applied using various frameworks such as the Reflective Agile Iterative Design (RAID) [65], phased development [47], and iterative design [19,48,50,56]. Participatory development with iterative approaches was shaped by the level of engagement, type of stakeholders, and reason for involvement.

#### Participatory Development (Domain 1)

The studies showed different levels of end users’ engagement and participation throughout the design and development process. The 14 (82%) of the 17 studies that applied an inclusive theory or approach involved people with IDs as primary stakeholders throughout the full development process to facilitate a full understanding of users’ perceptions, needs, and abilities [19,46-48,51,55-58,60,62-65]. In total, 2 (12%) of the 17 studies reported end user involvement in early-stage prototype testing to ensure that important usability and accessibility issues (eg, language use and button size) could be corrected [47,56]. Moreover, 3 (18%) of the 17 studies did not adopt a theoretical approach to guide inclusive development [59,61], and in 1 of these studies, the end user provided feedback only through informative interviews [50].

In total, 9 (53%) of the 17 studies reflected a design process that was collaborative with other key stakeholders such as family, care providers, and other professionals [19,50,51,56-58,61,62,64]. These stakeholders facilitated the studies by providing input in interviews about the needs of the target group or were involved as active participants in the development process [19,58]. Of these, 1 study described secondary users’ (eg, caregivers and parents) and tertiary users’ (eg, teachers) experiences with using the technology in addition to the use by the primary users (eg, people with IDs) [57]. The stakeholders were also included in the studies to gather important values and needs to shape the eHealth technologies, for example, by interviewing them to retrieve specific technical objectives [50] or operational requirements [61]. In another study, board members of an association representing most people with IDs were contacted as stakeholders to explain the specific needs that were not covered by the existing technological device [59].

#### Iterative Process (Domain 2)

Of the 17 studies, 13 (76%) studies mentioned an iterative process approach in which the end users or other stakeholders were involved in developing and improving eHealth technologies. In 11 (65%) of the 17 studies, iterations were performed with the stakeholders by gathering their feedback [19,46-48,56,58,60,62-65]. This was done by performing continuous evaluations [19,47,48,51,56,60,63,65]; creating and improving prototypes based on observations and design challenges identified by using the technology [46,58,62,64]; and gathering, refining, and validating user requirements [61]. Furthermore, 4 (24%) of the 17 studies did not mention iterative cycles during development [55,59], and 2 (12%) of the 17 studies suggested future iterations [50,57].

#### Value Specification and Value Proposition (Domains 3 and 4)

Regarding value specification, the included studies used various strategies to describe and identify their end users’ needs and values to create an optimum level of return. First, end users’ specific characteristics were identified, including their age; gender; literacy level; or syndromes and disorders such as cerebral palsy [56], Down syndrome [56,57], autism spectrum disorder [56], Fragile X syndrome [58], and Prader-Willi syndrome [64]. Second, existing definitions were used, for example, those of the American Psychiatric Association [66] and the American Association on Intellectual and Developmental Disabilities [51,65,67]. In total, 4 (24%) of the 17 studies described the end users’ characteristics and the values that were related to the cognitive ability to manage, for example, tasks switching [64] and the targeted end users’ exposure to, and the degree of (independent) use of, technology [46,50,61]. A total of 9 (53%) of the 17 studies reported the identification of other stakeholders in addition to the end users (eg, family, friends, teachers, support workers, health care professionals, communities, and coaches) [19,50,51,56-58,61,62,64,65].

The value proposition of the included studies differed depending on the kind of value that the developed technology could generate for potential end users. These studies mainly focused on nonfinancial values (eg, symptom control); in contrast, financial values (eg, profit) were not mentioned. Independent access to transport [47,51,55], social participation skills [46], and communication were found as examples of values for potential end users [50]. Development of the value proposition was, in most cases, based on findings or recommendations found in previous research [47,48,50,58,60,62-64] or end users’ demand for greater accessibility or needs ascertained from the researchers’ findings [57,59,65]. In total, 2 (12%) of the 17 studies reviewed the content and design of existing comparable technologies and based the development on these insights [56,58].

#### Technological Development and Design (Domain 5)

To translate the identified values into technology, 4 different steps in the development and design process were described in 10 (59%) of the 17 studies. First, technological requirements based on the values were identified and analyzed so that they could be applied in the technologies to match end users’ accessibility and usability [47,50,55,57]. Second, interaction design patterns, which are a formal way of documenting a solution to a common design problem, were specified and translated for implementation in the prototype [61]. Third, the technology was developed in 2 phases. During the first phase, the end users’ needs considering the desired design were ascertained and converted into a program of requirements in the second phase [19]. Finally, user requirements were translated into design requirements in 4 (24%) of the 17 studies, for example, by consulting the end users at the start of the project regarding their preferences [46,51,59,64].

Methods that were performed for specifying user requirements were as follows: interviews with stakeholders [56]; advisory group input [62]; surveys assessing needs, requirements, and use of the technology [57]; an environmental scan evaluating apps to determine the features that needed to be included [58]; or a reflective conversation with stakeholders in the problem context [65].

The development and use of prototypes, mock-ups, or test versions based on the identified requirements were mentioned in most studies, 16 (94%) out of 17 studies [19,46-48,50,51,56-65]. To adjust the development process to specific end user groups (ie, people with IDs), methods such as RAID allowed for an approach that linked prototyping with an approach that emphasized the use and importance of creating prototypes when working with individuals for whom abstraction of thought could be difficult [65]. Furthermore, a sensitive way of designing was used, offering the target group the opportunity to test the lo-fi and prototype versions, ensuring that the goals of the overall system could be met [47].

Prototypes were used to address and improve the usability of the developed technology, and usability tests were performed with (proposed) end users [19,48,50,51,56,58] and other stakeholders such as caregivers, parents, and coaches [51]. Technology workshop sessions were used to identify and consider usability issues [46]; questionnaires were administered to provide feedback on the use of the technology [64]; and expert reviews and usability evaluations with test interface sketches [61] were performed. The reasons mentioned for using these procedures included improving usability [19,46,47,50,51,56,58], improving accessibility [56], and identifying issues related to the technology such as the use of widget symbols and the need for community safety [47]. Only a few studies conducted field tests [57,58] or tests under real-life conditions [59]. Only 1 study did not mention usability testing with the prototypes [55].

#### Organization and External Context (Domains 6 and 7)

The included studies did not examine the changes needed within the organization after the introduction of technology. Moreover, the external context with conditions that could complicate adoption and spread was underrepresented, as these studies focused predominantly on the individual use of the technology and addressed organizational and external contexts only marginally. Only 1 study took place within a health care organization in which a shift in the caregivers’ approach from supply-driven care to client-centered care aimed at improving the personal strength of clients with disabilities was mentioned as a change needed [19].

#### Implementation and Evaluation (Domains 8 and 9)

Only 3 (18%) of the 17 studies addressed implementation from the start [19,47,65], and 1 study showed 2 implementation phases performed by the designers [47]. Future implementation of technologies received some attention in the recommendations of the studies. In total, 4 (24%) of the 17 studies stated that the design of the technology needed to be improved before it could be ready for future (iterative) testing and implementation in practice [48,50,56,65]. Another study engaged end users to participate in an app to develop more confidence and sustain independent participation and appropriation over time [46]. Further work required to examine strategies to promote access to the technology for people with IDs and to identify options for future iterations of the system was also mentioned [50].

For the evaluation phase, studies described the analysis and reported on whether the uptake of the technology was as intended by the developers [46,51,56,57,59-61,63,65]. Evaluations focused on several aspects: (1) users’ understanding of the content of the technology [60], (2) the use of the developed eHealth technology as intended [59,63], (3) independent use in the long term and its challenges [46,61], and (4) the integration and support of the technology in end users’ daily lives [57,65]. The impact of the technologies on the individual user was evaluated by measuring general outcomes such as increased independence [19,48,57], inclusion [50], confidence [48], and self-expression and socialization [65]. Other types of impact mentioned in the studies were related to topic-specific outcomes, such as better reducing the need to travel [58] and the ability to structure and support daily activities better [19].

## Discussion

### Principal Findings

This study was the first to review inclusive approaches used in eHealth design, development, and implementation processes for people with IDs and assessed 9 domains based on the CeHRes road map and the NASSS framework. Our findings showed that the domains participatory development, iterative process, and technological development and design use inclusive approaches that were applied reflectively and iteratively and based on participatory approaches including human- or user-centered design and participatory development. End users were involved primarily early in the process to ascertain their needs and during usability testing, whereas their and other stakeholders’ involvement was mostly lacking in later phases. The domains external context, organizational context, and the financial side of the value proposition were underrepresented in the literature because the focus was predominantly on the individual use of eHealth technologies. However, members of the target group rely on their (social) environment for care and support. Involving these key stakeholders in the ID sector during the design, development, and implementation phases and giving more attention to the underrepresented domains can improve the fit between the technology, end user, and context.

By combining the CeHRes road map and the NASSS framework for technologies within health care, we created a broad perspective regarding the design, development, and implementation processes of eHealth for people with IDs [32,68]. CeHRes road map describes clear development activities and elements, and the NASSS framework shows important preconditions for implementation [17,30]. The 9 identified domains can be used in every iteration to ensure and report stakeholders’ involvement in every domain of the process. Our study demonstrates the applicability of integrated frameworks and their potential to investigate and describe eHealth design, development, and implementation in future studies and supports the use of inclusive approaches in each domain. Reporting the process of inclusive design, development, and implementation along the 9 different domains of our integrated framework facilitates the sharing of experiences and knowledge about inclusive eHealth development and implementation.

The studies using inclusive approaches [19,46-48,51,55-58,​60,62-65] showed that specific problems experienced by people with IDs as end users (eg, difficult language use and usability issues) can be addressed only through a user-centered approach [17]. Notably, the included studies focused mainly on people with IDs as end users, and they often strongly depend on support persons (eg, caregivers) for access to and use of eHealth services. Therefore, future research should investigate the roles of support persons in relation to eHealth solutions and include them in the development and implementation processes [3]. This also raises the question of whether a universal design is a plausible goal. Persons with disabilities, particularly IDs, are a very heterogeneous group in which this goal could be difficult to reach [10]. However, when developing eHealth for people with IDs, it does provide access to a large group of people because elements such as accessibility, usability, cognitive capacity, digital skills, and low literacy are taken into account. In addition, our study showed that inclusive approaches go hand in hand with iterative processes, such as iterative design and RAID. These approaches allowed for improvements to the design by performing continuous evaluations. Iterations were identified mainly in the design and development processes of the included studies. However, by performing these iterations during the implementation, usability issues that emerge after implementation in practice can be addressed [17]. Altogether, the development, implementation, and evaluation overlap and are iterative rather than separate linear phases [69]. It is important to consider this overlap to reduce the misuse and abandonment of technologies and to ensure that important barriers to implementation are not overlooked during development [1]. We suggest that future eHealth trajectories consider at the start whom they need to involve and when to improve the use of, and access to, eHealth by people with IDs [1,18]. By performing iterations together with end users and stakeholders, a good fit between the technology, context, and users can be ensured [1].

This review further indicates that the organization and external context domains are less addressed in the included papers. A possible explanation is that the described technologies focused mainly on the individual use of eHealth applications, with the result that less attention was given to the organization and external context. Themes related to the organizational context (eg, capacity and readiness to innovate and changes in organizational routines) and themes related to the external context (eg, political context and legal issues) can influence implementation at the individual and organizational levels. In line with this, financial values, such as the profitability of technologies, were also addressed marginally. However, these are important preconditions for sustainable implementation and can facilitate the adoption and spread of the technology [30,32].

### Strengths and Limitations

This scoping review included a broad, comprehensive, and systematic search performed to identify and select both published and unpublished gray literature and peer-reviewed scientific literature. The search strategy did not include studies on the evaluation process of eHealth technology after the implementation phase, although this can provide useful and important information about implications for development and implementation. We suggest that future studies explore other literature, for example, on the evaluation of the effectiveness and feasibility of eHealth for people with IDs, to collect more evidence on the evaluation process of eHealth for people with IDs. Second, the wide variety of terminology presented a challenge for the formulation of the search strategy. The term eHealth is regularly used as an umbrella term with diverse definitions [36] and different focuses, with the term, in a broader sense, characterizing not only a technical development but also a state of mind to improve health care by using information and communication technology [37] and indicating cost-effectiveness and secure use of information and communications technologies [70]. In addition, different terms are used for inclusive approaches in design, development, and implementation processes such as participatory development and design and user-centered design, thereby complicating the identification of inclusiveness in these approaches. We attempted to create a complete picture of the literature and avoid bias as much as possible by applying a wide search strategy (Multimedia Appendix 1) developed with the assistance of an information expert. A more specific mention of inclusiveness in the design, development, and implementation process of eHealth helps to make inclusive eHealth research easier to identify.

### Conclusions

This scoping review has demonstrated the applicability of the integrated frameworks—the CeHRes road map and the NASSS framework—and the potential of the 9 identified domains to investigate and describe eHealth design, development, and implementation processes in future studies. Participatory development, an iterative process, and technological development are the primary domains that surfaced. Most studies showed end user involvement and iterations in the design and development phases, whereas only a few studies involved end users and iterative processes during the implementation phase. The external and organizational context domains, the financial side of the value proposition, and the application of inclusive approaches with stakeholders other than the end user received little attention. However, members of this target group specifically rely on their (social) environment for care and support. By paying more attention to these underrepresented domains and including key stakeholders further on in the process, the translational gap that exists between the developed technologies and user needs, capabilities, and context can be reduced. This study is the first step toward creating a better understanding of inclusive eHealth design, development, and implementation processes for people with IDs.

## Appendix

Multimedia Appendix 1 Search strategy.

Multimedia Appendix 2 Domains and components used for data extraction.

## Abbreviations

CeHRes: Centre for eHealth Research and Disease management
ID: intellectual disability
NASSS: Nonadoption, Abandonment, and challenges to the Scale-up, Spread, and Sustainability
NASSS-CAT: Nonadoption, Abandonment, and challenges to Scale-up, Spread, and Sustainability-Complexity Assessment Tool
PRISMA-ScR: Preferred Reporting Items for Systematic Reviews and Meta-Analyses extension for Scoping Reviews
RAID: Reflective Agile Iterative Design
