# Supplementary material for: Toward Inclusive Approaches in the Design, Development, and Implementation of eHealth in the Intellectual Disability Sector: Scoping Review
Source: J Med Internet Res. 2023 May 30;25:e45819. doi: 10.2196/45819 (PMC10265410; doi:10.2196/45819)
Supplement: Multimedia Appendix 1 [file jmir_v25i1e45819_app1.docx]

Appendix 1: Search strategy

**Definition Intellectual disability**: *“significant limitations both in intellectual functioning and in adaptive behaviour as expressed in conceptual, social, and practical adaptive skills”.*

(Deuel RK. Mental retardation: definition, classification, and systems of supports (10th ed). Pediatric Neurology. 2003 07/01/;29(1):80. doi: 10.1010/S0887-8994(03)00213-3.)

**Definition eHealth**: *“the use of technologies to improve health, well-being, and healthcare”.*

(van Gemert-Pijnen J, Kelders S, Kip H, Sanderman R. eHealth Research, Theory and Development. Milton Park, Oxfordshire: Taylor & Francis; 2018.)

**Definition design: “***a drawing or set of drawings showing how a building or product is to be made and how it will work and look.”* ([Cambridge Dictionary of American English](https://dictionary.cambridge.org/dictionary/english/design). Definition of ‘Design’. Accessed on 12/05/2021, retrieved from [DESIGN | English meaning - Cambridge Dictionary](https://dictionary.cambridge.org/dictionary/english/design))

**Definition development:** *"The systematic use of scientific, technical, economic and commercial knowledge to meet specific business objectives or requirements".* ([L. Al-Hakim](https://www.igi-global.com/affiliate/latif-al-hakim/296631/), [X. Wu](https://www.igi-global.com/affiliate/xiaobo-wu/296632/), [A. Koronios](https://www.igi-global.com/affiliate/andy-koronios/296633/) and [Y. Shou](https://www.igi-global.com/affiliate/yongyi-shou/296634/). (2016). Handbook of Research on Driving Competitive Advantage through Sustainable, Lean, and Disruptive Innovation. <https://www.igi-global.com/book/handbook-research-driving-competitive-advantage/142171>)

**Definition implementation:** *"a specified set of activities designed to put into practice an activity or program of known dimensions".* (D.L. Fixsen, S. Naoom, K. Blasé, R. Friedman & F. Wallace. (2005). Implementation Research: A Synthesis of the Literature. <http://ctndisseminationlibrary.org/PDF/nirnmonograph.pdf>)

1. Database: **PubMed**
   Search Strategy:

| **#** | **Query** |
| --- | --- |
| 1 | ("Intellectual Disability"[MeSH] OR "Persons with Mental Disabilities"[MeSH] OR "Developmental Disabilities"[MeSH] OR "Learning Disabilities"[MeSH:noexp] OR "Cognitive Dysfunction"[MeSH] OR "intellectual disab*"[Tiab] OR "intellectually disab*"[Tiab] OR "learning disab*"[Tiab] OR "developmental disab*"[Tiab] OR "developmentally disab*"[Tiab] OR "mental disab*"[Tiab] OR "mentally disab*"[Tiab] OR "mental retard*"[Tiab] OR "mentally retard*"[Tiab] OR "mental handicap*"[Tiab] OR "mentally handicap*"[Tiab] OR "mental deficien*"[Tiab] OR "mentally deficien*"[Tiab] OR "mental impair*"[Tiab] OR "mentally impair*"[Tiab]) NOT ("dement*"[Ti] OR "alzheimer*"[Ti] OR "parkinson"[Ti] OR "psychiatr*"[Ti] OR "injur*"[Ti]) |
| 2 | Telemedicine[MeSH] OR Telenursing[MeSH] OR Internet[MeSH] OR "Educational Technology"[MeSH] OR "Telecommunications"[MeSH] OR Programmed instruction as Topic[MeSH] OR "Computers"[Mesh] OR Cell Phones[MeSH] OR "Online Systems"[MeSH] OR "Computer-Assisted Instruction"[MeSH] OR "Self-help Devices"[MeSH] OR "Information Technology"[Mesh] OR "Electronic Health Records"[Mesh] OR "Patient Portals"[Mesh] OR "Computer Simulation"[Mesh] OR "Wearable Electronic Devices"[Mesh] |
| 3 | eHealth[tiab] OR e-health[tiab] OR "Electronic health"[tiab] OR Telehealth[tiab] OR Tele-health[tiab] OR Telemedicine[tiab] OR Tele-medicine[tiab] OR Telepractice[tiab] OR Tele-practice[tiab] OR Mhealth[tiab] OR M-health[tiab] OR Etherap*[tiab] OR E-therap*[tiab] OR "Electronic therap*"[tiab] OR Teletherap*[tiab] OR Tele-therap*[tiab] OR "Mobile health"[tiab] or m-health[tiab] OR mhealth[tiab] OR Ereferral*[tiab] OR E-referral*[tiab] OR "Electronic referral*"[tiab] OR Eprescri*[tiab] OR E-prescri*[tiab] OR Teleconsult*[tiab] OR Tele-consult*[tiab] OR Telepsychiatr* OR Tele-psychiatr* OR Telenursing[tiab] OR Tele-nursing[tiab] OR Telecare[tiab] OR Tele-care[tiab] OR Telemonitor*[tiab] OR Tele-monitor*[tiab] OR Telerehab*[tiab] OR Tele-rehab*[tiab] OR Telecommunication*[tiab] OR Tele-communication*[tiab] OR "Digital health"[tiab] OR "Information technology"[tiab] OR "Medical informatic*"[tiab] OR "Remote consult*"[tiab] OR Computer*[tiab] OR Microcomputer*[tiab] OR "Cell phone*"[tiab] OR Handheld[tiab] OR Mobile[tiab] OR Smartphone*[tiab] OR Iphone*[tiab] OR Tablet*[tiab] OR Ipad*[tiab] OR Internet[tiab] OR "Online system*"[tiab] OR Software[tiab] OR "User-Computer Interface"[tiab] OR "User interface"[tiab] OR Website*[tiab] OR Webpage*[tiab] OR Webinterface[tiab] OR "Web interface"[tiab] OR Ios[tiab] OR Android[tiab] OR "Electronic mail"[tiab] OR Email*[tiab] OR e-mail*[tiab] OR "Short message service*"[tiab] OR SMS[tiab] OR "Text messag*"[tiab] OR Multimedia[tiab] OR "Multimedia message*"[tiab] OR "Mobile application*"[tiab] OR "Mobile APP*"[tiab] OR "Electronic application*"[tiab] OR App[tiab] OR Apps[tiab] OR "Personal digital assistant*"[tiab] OR "Communications media"[tiab] OR Whatsapp[tiab] OR Skype[tiab] OR Social media[tiab] OR Podcast*[tiab] OR "Online education"[tiab] OR "Online training"[tiab] OR "Online instruction*"[tiab] OR "Educational technology"[tiab] OR "Programmed instruction*"[tiab] OR "Self-help device*"[tiab] OR "Assistive device*"[tiab] OR "Assisted device*"[tiab] OR "Serious gam*"[tiab] OR Wearable*[tiab] OR "Computer simulation*"[tiab] OR Virtual[tiab] OR "Virtual reality"[tiab] OR "Augmented reality"[tiab] OR "Computer-assisted therapy"[tiab] OR "Computer-assisted instruction*"[tiab] OR "Computer-assisted diagnos*"[tiab] OR Domotic*[tiab] OR "Electronic health record*"[tiab] OR Web-based intervention*[tiab] OR "Online intervention*"[tiab] OR "Remote monitor*"[tiab] OR "Remote patient monitor*"[tiab] OR Web-based[tiab] |
| 4 | (Develop*[tiab] OR Implement*[tiab] OR Design*[tiab]) OR Creat*[tiab] NOT (Design:[tiab]) |
| 5 | (#1) AND (#2 OR #3) AND (#4) AND ("english"[Language] AND (humans[Filter]) AND (1995:2021[pdat])) |

1. Database: **Embase**
   Search Strategy:

| **#** | **Query** |
| --- | --- |
| 1 | (exp *mental deficiency/ or *intellectual impairment/ or *learning disorder/ or exp *developmental disorder/ or *mentally disabled person/ or exp cognitive defect/ or (intellectual disab* or intellectually disab* or learning disab* or developmental disab* or developmentally disab* or mental disab* or mentally disab* or mental retard* or mentally retard* or mental handicap* or mentally handicap* or mental deficien* or mentally deficien* or mental impair* or mentally impair*).ti,ab,kw.) not (dement* or alzheimer* or parkinson or psychiatr* or injur*).ti. |
| 2 | exp telehealth/ or exp internet/ or educational technology/ or telecommunication/ or teleconference/ or exp telemetry/ or exp assistive technology/ or exp assistive technology device/ or computer assisted therapy/ or communication aid/ or exp mobile phone/ or exp mobile application/ or exp microcomputer/ or exp e-mail/ or exp information technology/ or exp multimedia/ or audiovisual aid/ or self help device/ or exp rehabilitation equipment/ or exp "dressing and undressing assistive device"/ or exp text messaging/ or exp text to speech technology device/ or exp electronic health record/ or exp mobile application/ or web browser/ or computer simulation/ or exp augmented reality/ or exp virtual reality/ or exp social media/ or exp smartphone/ or exp tablet computer/ or exp personal digital assistant/ or online system/ or head-mounted display/ or display system/ or wearable computer/ or smart glasses/ or virtual reality head mounted display/ or body area network/ or remote control/ or exp information technology device/ |
| 3 | eHealth/ or e-health/ or Electronic health/ or Telehealth/ or Tele-health/ or Telemedicine/ or Tele-medicine/ or Telepractice/ or Tele-practice/ or Mhealth/ or M-health/ or Etherap*/ or E-therap*/ or Electronic therap*/ or Teletherap*/ or Tele-therap*/ or Mobile health/ or m-health/ or mhealth/ or Ereferral*/ or E-referral*/ or Electronic referral*/ or Eprescri*/ or E-prescri*/ or Teleconsult*/ or Tele-consult*/ or Telepsychiatr*/ or Tele-psychiatr*/ or Telenursing/ or Tele-nursing/ or Telecare/ or Tele-care/ or Telemonitor*/ or tele-monitor*/ or Telerehab*/ or Tele-rehab*/ or Telecommunication*/ or Tele-communication*/ or Digital health/ or Information technology/ or Remote consult*/ or Computer*/ or Microcomputer*/ or Cell phone*/ or Handheld/ or Mobile/ or Smartphone*/ or Iphone*/ or Tablet*/ or Ipad*/ or Internet/ or Online system*/ or Software/ or User-Computer Interface/ or Website*/ or Webpage*/ or Web interface/ or Ios/ or Android/ or Electronic mail/ or Email*/ or Short message service*/ or SMS/ or Text messag*/ or Multimedia/ or Multimedia message*/ or Mobile application*/ or Mobile APP*/ or Electronic application*/ or App/ or Apps/ or Personal digital assistant*/ or Communications media/ or Whatsapp/ or Skype/ or Social media/ or Podcast*/ or Online education/ or Online training/ or Educational technology/ or Programmed instruction*/ or Self-help device*/ or Assistive device*/ or Assisted device*/ or Serious gam*/ or Wearable*/ or Computer simulation*/ or Virtual/ or Virtual reality/ or Augmented reality/ or Computer-assisted therapy/ or Computer-assisted instruction*/ or Computer-assisted diagnos*/ or Domotic*/ or electronic health record*.ti,ab,kw. |
| 4 | Develop*/ or Implement*/ or Design*/ or Creat* NOT Design:.ti,ab,kw |
| 5 | Search: (#1) AND (#2 OR #3) AND (#4) |
| 6 | limit 5 to (english language and yr="1995 -Current") |

1. Database: **APA PsycInfo**
   Search Strategy:

| **#** | **Searches** |
| --- | --- |
| 1 | (exp *intellectual development disorder/ or *delayed development/ or *developmental disabilities/ or *learning disabilities/ or *cognitive impairment/ or (intellectual disab* or intellectually disab* or learning disab* or developmental disab* or developmentally disab* or mental disab* or mentally disab* or mental retard* or mentally retard* or mental handicap* or mentally handicap* or mental deficien* or mentally deficien* or mental impair* or mentally impair*).ti,ab,id.) not (dement* or alzheimer* or parkinson or psychiatr* or injur*).ti |
| 2 | exp assistive technology/ or exp human technology interaction/ or exp "information and communication technology"/ or exp communication systems/ or exp telemedicine/ or exp computer assisted therapy/ or exp electronic health services/ or exp online therapy/ or exp teleconferencing/ or exp teleconsultation/ or exp telepsychiatry/ or exp telepsychology/ or exp telerehabilitation/ or exp computer applications/ or exp computer mediated communication/ or exp digital interventions/ or exp health care services/ or exp health knowledge/ or exp internet/ or exp telecommunications media/ or exp videoconferencing/ or exp mobile phones/ or exp mobile devices/ or exp telephone systems/ or exp smartphones/ or exp mobile applications/ or exp mobile application/ or exp wireless technologies/ or exp online therapy/ or exp mobile health/ or exp microcomputers/ or exp multimedia/ or exp communications media/ or exp audiovisual communications media/ or exp text messaging/ or exp electronic communication/ or exp electronic health records/ or exp Computer Software/ or exp Virtual Reality/ or exp augmented reality/ or exp virtual reality exposure therapy/ or exp social media/ or exp tablet computers/ or exp distance education/ or programmed instruction/ or computer assisted instruction/ or exp Computer Assisted Therapy/ or exp Computer Assisted Diagnosis/ or exp Websites/ or wearable devices/ or mobile technology/ or digital technology/ or simulation/ or computer simulation/ or simulation games.mp. [mp=title, abstract, heading word, table of contents, key concepts, original title, tests & measures, mesh] |
| 3 | (Develop*/ or Implement*/ or Design*/ or Creat*.mp.) not Design:.ti,ab,id. |
| 4 | S1 AND S2 AND S3 |
| 5 | Limit 4 to (english language and yr="1995-Current") |

# Database: **CINAHL** Search Strategy:

| **#** | **Searches** |
| --- | --- |
| S1 | (MH "Mentally Disabled Persons") OR (MH "Developmental Disabilities") OR (MH "Intellectual Disability+") OR (MH "Learning Disorders") OR TI ("intellectual disab*" OR "intellectually disab*" OR "learning disab*" OR "developmental disab*" OR "developmentally disab*" OR "mental disab*" OR "mentally disab*" OR "mental retard*" OR "mentally retard*" OR "mental handicap*" OR "mentally handicap*" OR "mental deficien*" OR "mentally deficien*" or "mental impair*" OR "mentally impair*") OR AB ("intellectual disab*" OR "intellectually disab*" OR "learning disab*" OR "developmental disab*" OR "developmentally disab*" OR "mental disab*" OR "mentally disab*" OR "mental retard*" OR "mentally retard*" OR "mental handicap*" OR "mentally handicap*" OR "mental deficien*" OR "mentally deficien*" or "mental impair*" OR "mentally impair*") NOT TI (dement* or alzheimer* or parkinson or psychiatr* or injur*) |
| S2 | (MH "Telecommunications+") OR (MH "Telehealth+") OR (MH "Telerehabilitation") OR (MH "Internet+") OR (MH "Social Media+") OR (MH "World Wide Web+") OR (MH "Educational Technology") OR (MH "Assistive Technology") OR (MH "Programmed Instruction+") OR (MH "Computer Assisted Instruction") OR (MH "Computers and Computerization+") OR (MH "User-Computer Interface+") OR (MH "Cellular Phone+") OR (MH "Text Messaging+") OR (MH "Online Systems") OR (MH "Assistive Technology Devices+") OR (MH "Information Technology+") OR (MH "Electronic Health Records+") OR (MH "Wearable Sensors+") |
| S3 | TI ("Computers and Computerization" OR "Educational Technology" OR "Telehealth" OR "eHealth" OR "Teleconferencing" OR "Internet" OR "Telemedicine" OR "Telenursing" OR "Telepsychiatry" OR "Telenutrition" OR "Teledentistry" OR "Telerehabilitation" OR "Telecommunications" OR "Communications Media" OR "Email" OR "Interactive Voice Response Systems" OR "Telephone" OR "Text Messaging" OR "Videoconferencing" OR "Wireless Communications" OR "Electronic Health Records" OR "Patient Portals" OR "Mobile Health Units" OR "Remote Consultation" OR "Software" OR "Mobile Applications" OR "Multimedia" OR "Webcasts" OR "Virtual Reality" OR "Virtual Reality Exposure Therapy" OR "Augmented Reality" OR "Computer Simulation" OR "Text Messaging" OR "Instant Messaging" OR "Smartphone" OR "Computers, Hand-Held" OR "Computers, Portable" OR "Microcomputes" OR "Social Media" OR "Cellular Phone" OR "Digitizers" OR "Voice Recognition Systems" OR "Keyboards" OR "Mouse Computer" OR "Scanners" OR "Computer Input Devices" OR "Online Education" OR "Programmed Instruction" OR "Computer Assisted Instruction" OR "Online Systems" OR "Therapy, Computer Assisted" OR "Diagnosis, Computer Assisted" OR "Assistive Technology Devices" OR "Ambulation Aids" OR "Communication Aids for Disabled" OR "Wearable Sensors" OR "Accelerometers" OR "Smart Glasses" OR "User-Computer Interface" OR "World Wide Web Applications" OR "Website Development" OR "Video Games" OR "Games") OR AB ("Computers and Computerization" OR "Educational Technology" OR "Telehealth" OR "eHealth" OR "Teleconferencing" OR "Internet" OR "Telemedicine" OR "Telenursing" OR "Telepsychiatry" OR "Telenutrition" OR "Teledentistry" OR "Telerehabilitation" OR "Telecommunications" OR "Communications Media" OR "Email" OR "Interactive Voice Response Systems" OR "Telephone" OR "Text Messaging" OR "Videoconferencing" OR "Wireless Communications" OR "Electronic Health Records" OR "Patient Portals" OR "Mobile Health Units" OR "Remote Consultation" OR "Software" OR "Mobile Applications" OR "Multimedia" OR "Webcasts" OR "Virtual Reality" OR "Virtual Reality Exposure Therapy" OR "Augmented Reality" OR "Computer Simulation" OR "Text Messaging" OR "Instant Messaging" OR "Smartphone" OR "Computers, Hand-Held" OR "Computers, Portable" OR "Microcomputers" OR "Social Media" OR "Cellular Phone" OR "Digitizers" OR "Voice Recognition Systems" OR "Keyboards" OR "Mouse Computer" OR "Scanners" OR "Computer Input Devices" OR "Online Education" OR "Programmed Instruction" OR "Computer Assisted Instruction" OR "Online Systems" OR "Therapy, Computer Assisted" OR "Diagnosis, Computer Assisted" OR "Assistive Technology Devices" OR "Ambulation Aids" OR "Communication Aids for Disabled" OR "Wearable Sensors" OR "Accelerometers" OR "Smart Glasses" OR "User-Computer Interface" OR "World Wide Web Applications" OR "Website Development" OR "Video Games" OR "Games") |
| S4 | TI ((Develop*) OR (Implement*) OR (Design*) OR (Creat*) NOT (Design:)) OR AB ((Develop*) OR (Implement*) OR (Design*) OR (Creat*) NOT (Design:)) |
| S5 | S1 AND (S2 OR S3) AND S4 |
| S6 | **Limiters** - Published Date: 19950101-20211231  **Narrow by Language:**- dutch/flemish  **Narrow by Language:**- english |

# Database: **Cochrane Library** Search Strategy:

| **#** | **Searches** |
| --- | --- |
| 1 | ([mh “Intellectual Disability”] OR [mh “Learning Disabilities”] OR [mh “Developmental Disabilities”] OR [mh “Cognitive Dysfunction”] OR Intellectual NEXT disab* OR Intellectually NEXT disab* OR Learning NEXT disab* OR Developmental NEXT disab* OR Developmentally NEXT disab* OR Mental NEXT disab* OR Mentally NEXT disab* OR Mental NEXT retard* OR Mentally NEXT retard* OR Mental NEXT handicap* OR Mentally NEXT handicap* OR Mental NEXT deficien* OR Mentally NEXT deficien* or mental NEXT impair* OR mentally NEXT impair*):ti,ab,kw NOT (dement* or alzheimer* or parkinson or psychiatr* or injur*):ti |
| 2 | (eHealth OR “Electronic health” OR “Electronic healthcare record” OR Telehealth OR Telemedicine OR Tele-medicine OR Telepractice OR Mhealth OR Etherap* OR E-therap* OR “Mobile health” OR Ereferral* OR “Electronic referral” OR “Electronic referrals” OR Eprescri* OR Teleconsult* OR Telenursing OR “Digital health” OR Software OR Telecommunication* OR Tele-communication* OR “Remote consultation” OR “Remote consultations” OR Podcast* OR Virtual OR “Virtual reality” OR “Virtual reality therapy” OR SMS OR App* OR Application* OR “Short message service” OR “Short message services” OR “Text message” OR “Text messages” OR “Text messaging” OR “Multimedia message” OR “Multimedia messages” OR Email* OR “Social media” OR Mobile OR “Cell phone” OR “Cell phones” OR Smartphone* OR Ios OR Android OR Ipad* OR Iphone* OR Tablet* OR Computer* OR Microcomputer* OR “Online education” OR “Online training” OR “Personal digital assistant” OR Internet OR “Communications media” OR “Programmed instruction” OR “Programmed instructions” OR Handheld* OR “Mobile application” OR “Mobile applications” OR “Mobile app” OR “Mobile apps” OR “Electronic application” OR “Electronic applications” OR “Electronic app” OR “Online system” OR “Online systems” OR “Computer-assisted instruction” OR “Self-help device” OR “Self-help devices” OR “Assistive device” OR “Assistive devices” OR “Assisted device” OR “Assisted devices” OR Telecare OR Telemonitoring OR Wearable*):ti,ab,kw |
| 3 | ((Develop* OR Implement* OR Design* OR Creat*) NOT (Design:)):ti,ab,kw |
| 4 | (#1) AND (#2) AND (#3) |
| 5 | "#4 - (#1) AND (#2) AND (#3)" with Cochrane Library publication date Between Jan 1995 and Jun 2021 |

1. Database(s): **Web of Science**
   Search Strategy:

| **#** | **Searches** |
| --- | --- |
| 1 | TOPIC: ("Intellectual disab*" OR "Intellectually disab*" OR "Learning disab*" OR "Developmental disab*" OR "Developmentally disab*" OR "Mental disab*" OR "Mentally disab*" OR "Mental retard*" OR "Mentally retard*" OR "Mental handicap*" OR "Mentally handicap*" OR "Mental deficien*" OR "Mentally deficien*" or "mental impair*" OR "mentally impair*") NOT TITLE: (dement* or alzheimer* or parkinson or psychiatr* or injur*) |
| 2 | (eHealth OR e-health OR "Electronic health" OR Telehealth OR Tele-health OR Telemedicine OR Tele-medicine OR Telepractice OR Tele-practice OR Mhealth OR M-health OR Etherap* OR E-therap* OR "Electronic therap*" OR "Mobile health" OR Ereferral* OR E-referral* OR "Electronic referral*" OR Eprescri* OR E-prescri* OR Teleconsult* OR Tele-consult* OR Telenursing OR Tele-nursing OR "Digital health" OR Digihealth OR Software OR Telecommunication* OR Tele-communication* OR "Remote consultation*" OR Podcast* OR Virtual OR "Virtual reality" OR SMS OR app OR Apps OR "Short message service*" OR "Text messag*" OR "Multimedia message*" OR Facebook OR Email* OR "Social media" OR Mobile OR "Cell phone*" OR Smartphone* OR Ios OR Android OR Ipad* OR Iphone* OR Tablet* OR Computer* OR "Online education" OR "Online training" OR "Personal digital assistant*" OR Internet OR "Communications media" OR "Programmed instruction*" OR Computers OR Handheld OR "Mobile application*" OR "Electronic application*" OR "Cell phones" OR "Online system*" OR "Computer-assisted instruction*" OR "Computer-assisted diagnos*" OR "Self-help device*" OR "Assistive device*" OR "Assisted device*" OR Telecare OR Tele-care OR Telemonitoring OR Tele-monitoring) |
| 3 | (Develop* OR Implement* OR Design* OR Creat*) |
| 4 | (S1 AND S2 AND S3) |
| 5 | S4 AND 1995-2021 AND English |

# Database: **Google Scholar** Search Strategy:

| **#** | **Searches** |
| --- | --- |
| 1 | "Intellectual disability" "eHealth development" |
| 2 | "Intellectual disability" "eHealth implementation" |
| 3 | "learning disability" "eHealth development" |
| 4 | "learning disability" "eHealth implementation" |
| 5 | "developmental disability" "eHealth development" |
| 6 | "mental disability" "eHealth implementation" |
| 7 | "mentally disabled" "eHealth development" |
| 8 | "mentally disabled" "eHealth implementation" |
| 9 | "mentally handicapped" "eHealth development" |
| 10 | "mental impairment" "eHealth implementation" |
| 11 | "cognitive impairment" "eHealth development" |
| 12 | "cognitive impairment" "eHealth implementation" |
| 13 | "intellectual disability" "e-Health development" |
| 14 | "intellectual disability" "e-Health implementation" |
| 15 | "learning disability" "e-Health implementation" |
| 16 | "learning disability" "e-Health development" |
| 17 | "developmental disability" "e-Health development" |
| 18 | "developmental disability" "e-Health implementation" |
| 19 | "mental disability" "e-Health implementation" |
| 20 | "mental disability" "e-Health development" |
| 21 | "mentally disabled" "e-Health development" |
| 22 | "mental impairment" "e-Health development" |
| 23 | "cognitive impairment" "e-Health implementation" (2) |
| 24 | "cognitive impairment" "e-Health development" |
| 25 | "Intellectual disability" "Telehealth development" (3) |
| 26 | "Intellectual disability" "Telehealth implementation" |
| 27 | "learning disability" "Telehealth development" |
| 28 | "learning disability" "Telehealth implementation" |
| 29 | "developmental disability" "Telehealth implementation" |
| 30 | "mental disability" "Telehealth development" |
| 31 | "mental disability" "Telehealth implementation" |
| 32 | "mentally disabled" "Telehealth development" |
| 33 | "mentally disabled" "Telehealth implementation" |
| 34 | "mental impairment" "Telehealth development" |
| 35 | "mental impairment" "Telehealth implementation" |
| 36 | "cognitive impairment" "Telehealth development" |
| 37 | "cognitive impairment" "Telehealth implementation" |
| 38 | "Intellectual disability" "Telemedicine development" |
| 39 | "Intellectual disability" "Telemedicine implementation" |
| 40 | "learning disability" "Telemedicine development" |
| 41 | "learning disability" "Telemedicine implementation" |
| 42 | "developmental disability" "Telemedicine development" |
| 43 | "developmental disability" "Telemedicine implementation" |
| 44 | "mental disability" "Telemedicine implementation" |
| 45 | "mentally disabled" "Telemedicine development" |
| 46 | "mentally disabled" "Telemedicine implementation" |
| 47 | "mentally handicapped" "Telemedicine development" |
| 48 | "mentally handicapped" "Telemedicine implementation" |
| 49 | "mental impairment" "Telemedicine implementation" |
| 50 | "cognitive impairment" "Telemedicine development" |
| 51 | "cognitive impairment" "Telemedicine implementation" |

# Database: **Google Scholar** Search Strategy:

| **#** | **Searches** |
| --- | --- |
| 1 | "ehealth implementatie" "verstandelijke beperking" filetype:pdf |
| 2 | "e-health implementatie" "verstandelijke beperking" filetype:docx |
| 3 | "ehealth ontwikkeling" "verstandelijke beperking" filetype:pdf |
| 4 | "e-health ontwikkeling" "verstandelijke beperking" filetype:docx |
| 5 | "telehealth implementatie "verstandelijke beperking" filetype:pdf |
| 6 | "telehealth implementatie "verstandelijke beperking" filetype:docx |
| 7 | "telehealth ontwikkeling "verstandelijke beperking" filetype:pdf |
| 8 | "telehealth ontwikkeling "verstandelijke beperking" filetype:docx |
| 9 | "telemedicine implementatie "verstandelijke beperking" filetype:pdf |
| 10 | "telemedicine implementatie "verstandelijke beperking" filetype:docx |
| 11 | "telemedicine ontwikkeling "verstandelijke beperking" filetype:pdf |
| 12 | "telemedicine ontwikkeling "verstandelijke beperking" filetype:docx |

1. **Terms for grey literature (Dutch)**

| **Verstandelijke beperking** | **eHealth** | **Design, ontwikkeling & implementatie** |
| --- | --- | --- |
| Verstandelijk beperkt | e-Health | Ontwikkelen |
| Verstandelijk gehandicapt | Electronic health | Ontwikkeld |
| Verstandelijke handicap | Telehealth | Implementatie |
| Verstandelijke ontwikkelingsstoornis | Tele-health | Implementeren |
| Geestelijke handicap | Telemedicine | Implementeert |
| Geestelijk gehandicapt | Tele-medicine | Design |
| Geestelijk beperkt | Telepractice | Creatie |
| Intellectuele beperking | Tele-practice | Creëert |
| Intellectueel beperkt | Telecare | Creëren |
| Intellectueel gehandicapt | Tele-care |  |
| Intellectuele handicap | Telemonitoring |  |
| Intellectuele stoornis | Tele-monitoring |  |
| Mentale handicap | Telenursing |  |
| Mentale retardatie | Tele-nursing |  |
| Mentale beperking | Telecommunicatie |  |
| Cognitieve beperking | Tele-communcatie |  |
| Cognitieve handicap | Teleconsultatie |  |
| Ontwikkelingsstoornis | Tele-consultatie |  |
|  | Mhealth |  |
|  | M-health |  |
|  | Digital health |  |
|  | Wearable |  |
|  | Domotica |  |
|  | Virtual reality |  |
|  | Augmented reality |  |
|  | Serious game |  |
|  | App |  |
