# Supplementary material for: Toward Inclusive Approaches in the Design, Development, and Implementation of eHealth in the Intellectual Disability Sector: Scoping Review
Source: J Med Internet Res. 2023 May 30;25:e45819. doi: 10.2196/45819 (PMC10265410; doi:10.2196/45819)
Supplement: Multimedia Appendix 2 [file jmir_v25i1e45819_app2.docx]

Appendix 2: Domains and components for data extraction

| **Domain** | **Components** | **Explanation** |
| --- | --- | --- |
| ***Participatory design/research:***  *Approach to design/research attempting to actively involve all stakeholders in the design/research process to help ensure the result meets their needs and is usable.* | Co-creation | Stakeholder participation is essential. Stakeholders’ involvement spans the full development process. Stakeholders can be considered as actors that have different roles in the development of eHealth technologies, from ideation to evaluation. Through their roles in identifying needs, or specifying critical issues for design and implementation, they help to create the technology. |
|  | Multidisciplinary Project Management | Multidisciplinary project management facilitates the cooperation between those who are responsible for producing the technology (e.g. technical designers and health care professionals) and those who participate to ensure that eHealth technologies fit in with the needs and values (e.g. end users and health care insurers/payers). |
|  | Including stakeholders' perspective | Different methods should be used to gather information from the stakeholders and context to continuously include their perspectives in the activities and outcomes of the development, implementation and evaluation of the technology. |
| ***Iterative process:***  *Continuous evaluation of the technology needs to be interwoven with all stages in the development process.* | Continuous evaluation | The created technology need to be evaluated continuously. |
|  | Checking whether outcomes of previous phases are accounted for | During the whole process of development and implementation it should be checked whether the outcomes of previous phases have been accounted for in the current phase and that the outcomes of all phases are related to each other. |
| ***Value specification:***  *Creating the optimum level of return for all stakeholders involved by identifying, analysing, gathering and mapping their values.* | End users | The person who in the end uses or is intended to ultimately use the technology. |
|  | Condition/illness | The condition/illness of the end users should be clearly defined, also think of comorbidity, variable manifestations that can occur and the uncertain course of the illness/ condition. These factors could affect the ability to benefit from the technology. |
|  | Sociocultural factors | The sociocultural situation of the end users could affect the ability to benefit from the technology. By mapping the social or cultural factors of the end users these can be taken into account when developing and implementing the technology. |
|  | Stakeholder identification | Anyone who affects or is affected by the technology should be identified. |
|  | Stakeholder analysis | It should be clear what the tasks and roles of the stakeholders are in order to identify the key stakeholders. Next to the end users, other people might have to work with the technology. It is important to identify these people and what changes it will bring for them (e.g. staff, ICT & carers). |
|  | Value identification | By identifying the values from all involved (key-)stakeholders, it can be clarified what the added value of the technology should be according to them. A value map can be used to prioritize and categorize the identified values. |
| ***Value proposition****: Think about what kind of value the technology might generate for different groups of people. (‘Value’ can be financial, such as profit, or non-financial, such as control of symptoms)* | Business model | By creating a business model the idea for the technology can be defined and easily communicated, moreover it is a plan for the developer on how to make profit from it. |
|  | Supply- and demand model | Identify the interaction between the seller/supplier of the technology and the buyer/demander. The law of supply and demand defines the relationship between the price of the technology and the willingness of people to either buy or sell it. |
|  | Ownership | The ownership of the technology needs to be accounted for, think of where the support of the technology embedded and who is accountable for the service and contact with the supplier. |
| ***Technological development and design:***  *Think about the technology (e.g. a tool or piece of software), and how it might affect care.* | Technology requirements | The identified stakeholder values should be translated into specific technological requirement, including material properties, that state what is required from the technology. |
|  | Prototyping (lo-fi & hi-fi) | Both low-fidelity and high-fidelity prototypes of the technology have to be developed, so they can be tested before further development. |
|  | Usability tests | Usability tests of the prototypes have to be conducted with end users, experts and possibly other involved (key-)stakeholders. These usability tests provide input on further development of the technology and what knowledge is needed for use. |
| ***Organisation***:  *Think about the (healthcare) organisation an what changes are needed and what changes for them after the technology is introduced.* | Capacity and readiness to innovate | The organisation’s capacity and readiness to take on technological innovations. Think of the ICT structure and acceptance of staff. |
|  | Nature of adoption- and or funding decision | The organisation has to commission/purchase the technology. Is there a department that deals with this, who has to approve the purchase? |
|  | Change in organisational routines | The work needed to plan, implement and monitor change should be identified to be able to change existing organisational routines. |
| ***External context:***  *Conditions that could complicate adoption and spread of the technology.* | Political/policy context | What is the political context towards the technology? How does this influence development or implementation? |
|  | Regulatory/legal issues | Are there any legal issues concerning the technology? How can these legal issues be addressed? |
|  | Professional bodies | A professional body is an organisation with individual members practicing a profession or occupation in which the organisation maintains an oversight of the knowledge, skills, conduct and practice of that profession or occupation. What is the attitude of the professional bodies an patient organisations towards the technology? |
|  | Interorganisational networking | Introduction of the technology/innovation could be threatened by external changes that impact on the organisation, those changes should be identified and addressed. |
| ***Implementation:***  *A plan with a set of conditions or activities designed to put into action to start to use a technology in practice.* | Implementation accounted for from start | The conditions for implementation should be taken into account from the start of the project. Potential implementation issues such as limited resources (e.g. time and money) or personal drawbacks (e.g. motivation and anxieties) should be identified. |
|  | Determine activities for implementation plan | Determine concrete activities to implement the technology in practice. Think of appointing ambassadors and giving presentations. Using the business model, input of the stakeholders and implementation theory (in this the NASSS framework) to create a plan to make sure that the technology is introduced and used in practice in the long term. |
| ***Evaluation:***  *Understanding the relative benefits and costs of the technology in context of the proposed implementation.* | Determine impact on context and stakeholders | The impact of the technology on the context and stakeholders should be determined, based on the values from the stakeholders and value proposition. |
|  | Analyse uptake of technology | The uptake of the technology should be analysed in terms of adoption or use of the technology by predetermined users and implementation, and use within the intended context. |
